# Predicting the suitable cultivation areas of breadfruit crops *Artocarpus altilis* (Moraceae) under future climate scenarios in Central Java, Indonesia

**DOI:** 10.3389/fpls.2024.1363153

**Published:** 2024-04-30

**Authors:** Suyud Warno Utomo, Fatma Lestari, Andrio Adiwibowo, Fatmah, Micah R. Fisher, Hafizha Ilma Qadriina

**Affiliations:** ^1^ School of Environmental Science, Universitas Indonesia, Jakarta, Indonesia; ^2^ Department of Occupational Health and Safety, Faculty of Public Health, Universitas Indonesia, Depok, West Java, Indonesia; ^3^ Disaster Risk Reduction Center, Universitas Indonesia, Depok, West Java, Indonesia; ^4^ Disaster Management Study Program, School of Environmental Science, Universitas Indonesia, Jakarta, Indonesia; ^5^ East-West Center, University of Hawaii, Honolulu, HI, United States

**Keywords:** breadfruit, Central Java, RCP 2.6, RCP 8.5, species distribution modeling

## Abstract

*Artocarpus altilis*, commonly known as breadfruit, is a potential crop adapted to a wide variety of climates and widely spread, including in Indonesia. However, information on how this species can adapt to climate change, in particular in Central Java, is still limited. In Indonesia, Central Java is the center for cultivation areas for many crop species to support the 145 million people living on Java Island. One of the potential crops being developed in Central Java is breadfruit. To assess the suitable cultivation areas for breadfruit, species distribution modeling (SDM) was used to predict the current and future (2050–2070) distribution of breadfruit. Two climate change scenarios, including optimistic RCP2.6 and pessimistic RCP8.5 models, were considered to represent future climate change impacts. Based on the results for both optimistic and pessimistic scenarios, the breadfruit’s suitable cultivation areas will expand eastward. Implementing a mitigation climate change scenario and limiting the temperature increase to only 1°C under RCP2.6 will provide 270.967 km^2^ more of suitable cultivation areas for breadfruit in 2050 and 133.296 km^2^ in 2070. To conclude, this study provides important information on the status and potential cultivation areas for breadfruit, mainly in the Southeast Asia region. The identification of suitable areas will guide land conservation for breadfruit to support food security in this region.

## 1 Introduction

*Artocarpus* (around 70 species) is a plant genus of Moraceae native to South and Southeast Asia, with Borneo serving as a diversification hotspot. The genus contains numerous well-known medium to large tropical tree species. One of the well-known species is breadfruit [*Artocarpus altilis* (Parkinson ex F.A. Zorn) Fosberg]. This species is a horticultural plant (Rahmah and Waluyo, 2019). with nutritional properties similar to rice, and it is sometimes used as a substitute for rice in some regions of the world (USDA, 2018a, b). Breadfruit trees, in addition to being a prolific producer of nutritious fruits, have the potential not only to adapt, but also to thrive in conditions where other crops would perish. This plant is a staple food in the Pacific Belt and an important component of socio-cultural rituals and the medical system. Under present production standards, breadfruit growing delivers numerous environmental benefits. Breadfruit tree crops in general have been shown to provide numerous benefits, which are amplified in agroforestry settings (Barthel et al., 2013). Agroforestry can renew the soil’s litter layer, improve and maintain soil quality, retain soil nutrients, and reduce soil erosion (Gao et al., 2014; Langston and Lincoln, 2018). *Artocarpus* is an important staple crop that has been grown for centuries throughout Oceania under agroforestry systems. The crop’s long history and significance are reflected in language, art, and cultures. Breadfruit was recently included in Annex 1 of the International Treaty on Plant Genetic Resources (ITPGR) as one of 35 crops identified for their importance for food security and interdependence, and it is classified as a priority crop by the Global Crop Diversity Trust (http://www.croptrust.org/main/lprioritycrops.php) (Jones et al., 2012).

However, the recent trend toward deforestation, climate changes, and the inclusion of non-traditional cuisines has reduced breadfruit dependency in many Oceanian regions and influence either the breadfruit productivity or nutrient content (Erland et al., 2023). As reliance on bread fruit declines, the threat of genetic erosion grows, and many of these geographically confined cultivars face extinction. Agroforestry land uses are required for sustainable breadfruit production. In fact, current land use change has resulted in the rapid growth of deforestation due to settlement development, which has threatened and reduced the land available for agroforestry practices (Munjeb et al., 2020; Shapla et al., 2022). Deforestation can affect the breadfruit crops in indirect way through climate change impacts. Lima et al. (2022) predicted that climate change following land use changes and deforestation threatens native potential crop species as can be seen in Brazil. Climate change following deforestation is projected to cause a crop species to lose an average of 65% of its original environmentally suitable habitat (Gomes et al., 2019). According to the IPCC study (Tuddenham and Robert, 2022), human activities raise global temperatures by 1 ^0^C over preindustrial levels. Climate change (Ma and Sun, 2018) affects crop species distribution by altering climate parameters such as temperature seasonality, yearly precipitation, and annual mean temperature, resulting in abnormally dry months. The climate has an important role in limiting crop species spread by affecting the life cycle of the crop species by lowering or increasing temperature, precipitation, and wind speed factors. These changes can have an immediate impact on crop species ecology or induce population declines, which can lead to extinction (Masson-Delmotte et al., 2019). Climate change may pose an irrevocable threat to fauna and flora, as well as humans, in the long run.

In Indonesia, breadfruits are distributed all over the country. It is known as a versatile crop since all parts of breadfruit are consumable and can be used by humans (Estalansa et al., 2018). In Indonesia, breadfruit crops were also cultivated within agroforestry systems (Elevitch and Ragone, 2018; Adinugraha et al., 2021). Breadfruit is an important crop since its production is increasing from 35,435 metric tons to 92,014 in just within seven years, covering 13,359 ha. Areas in Indonesia used for breadfruit cultivations to deal with the food security issues covering Sumatra Island, including South Sumatera, Lampung, and Jambi, and Java Island, including West, Central, East Java, D.I Yogyakarta, East Kalimantan, and eastern Indonesia, including East Nusa Tenggara and South Sulawesi (Widowati, 2009). One of the areas with potential in Indonesia for growing breadfruit crops is Central Java. Currently, the existence of agroforestry and crop species in Indonesia is threatened by rapid climate change (Santika et al., 2017; Kemen et al., 2019). One of the areas in Indonesia that is currently threatened by rapid climate change is Java Island (Higginbottom et al., 2018). Central Java is one of the areas on Java Island known for its agroforestry potential and diverse crop species (Rozaki et al., 2021), as well as for breadfruit agroforestry (Purwaningsih et al., 2020), which is recently threatened by climate change. This climate change has the potential to threaten the existence of breadfruit crops and food security. The current study on breadfruit in Indonesia and Java Island focuses mainly on diversity aspects (Rahmah and Waluyo, 2019) (Estalansa et al., 2018), and growth performance (Adinugraha et al., 2021), yet a study on how climate change can impact the potential adaptation of breadfruit is still limited.

Currently, there are a growing variety of methods for estimating habitat appropriateness and suitability, known as species distribution modeling (SDM), including MaxEnt (maximum entropy), BIOCLIM, DOMAIN, generalized additive model (GAM), GLM, and BIOMAPPER. Each tool is unique, with its own set of advantages and disadvantages. According to Marcer et al. (2013), several advantages of SDM include the need for only species presence data, the capacity to run with a limited quantity of data, the high accuracy of prediction results, the high reproducibility, and the ability to predict the most discriminating bioclimatic factors (Fois et al., 2018).

In Indonesia, SDM has been used to estimate several plant species. SDM has been used to simulate *Baccaurea angulata* Merr. or ‘Belimbing Dayak,’ an underutilized fruit by indigenous people in Kalimantan. This species has the potential to be exploited as both an edible fruit and a therapeutic plant. Unfortunately, the conversion of forests to oil palm and rubber plantations has affect climatic variables and reduces the habitat of *B. angulata*. The findings indicate that four bioclimatic variables, namely solar radiation in October, altitude, precipitation in the warmest quarter, and slope, are important in determining *B. angulata’s* suitable environment. The location of acceptable habitat for *B. angulata* corresponds to its current distribution. The potential appropriate region was substantially larger than the current distribution of *B. angulata* in Kalimantan. West Kalimantan and South Kalimantan were found as the most suitable places in this study. They comprised sections of Sambas, Landak, Sanggau, Sekadau, and Bengkayang in West Kalimantan Provinces, and Tanah Laut and Banjar in South Kalimantan Provinces. It is shown that the SDM was extremely accurate and informative for *B. angulata* habitat suitability and potential distribution. The anticipated model of appropriate sites can be used for *B. angulata* management, monitoring, cultivation, and future conservation (Gunawan et al., 2021). Then SDM method has potential to be used to assess the potential cultivation areas for breadfruit.

Despite the potential of breadfruit agroforestry, particularly in Central Java, there is very little information on this crop species’ potential cultivation areas and how it is whether threatened or supported by current climate changes. This information is very important considering there are 145 million people living on Java Island, and the food security of the people depends on the available crops. Here, we used a SDM approach to (1) quantify potential breadfruit cultivation areas and then (2) assess potential cultivation areas that were impacted by climate change. The results will contribute significantly to sustaining the agroforestry practices of this species over the long term.

## 2 Materials and methods

### 2.1 Description of the study area

**Figure 1:**
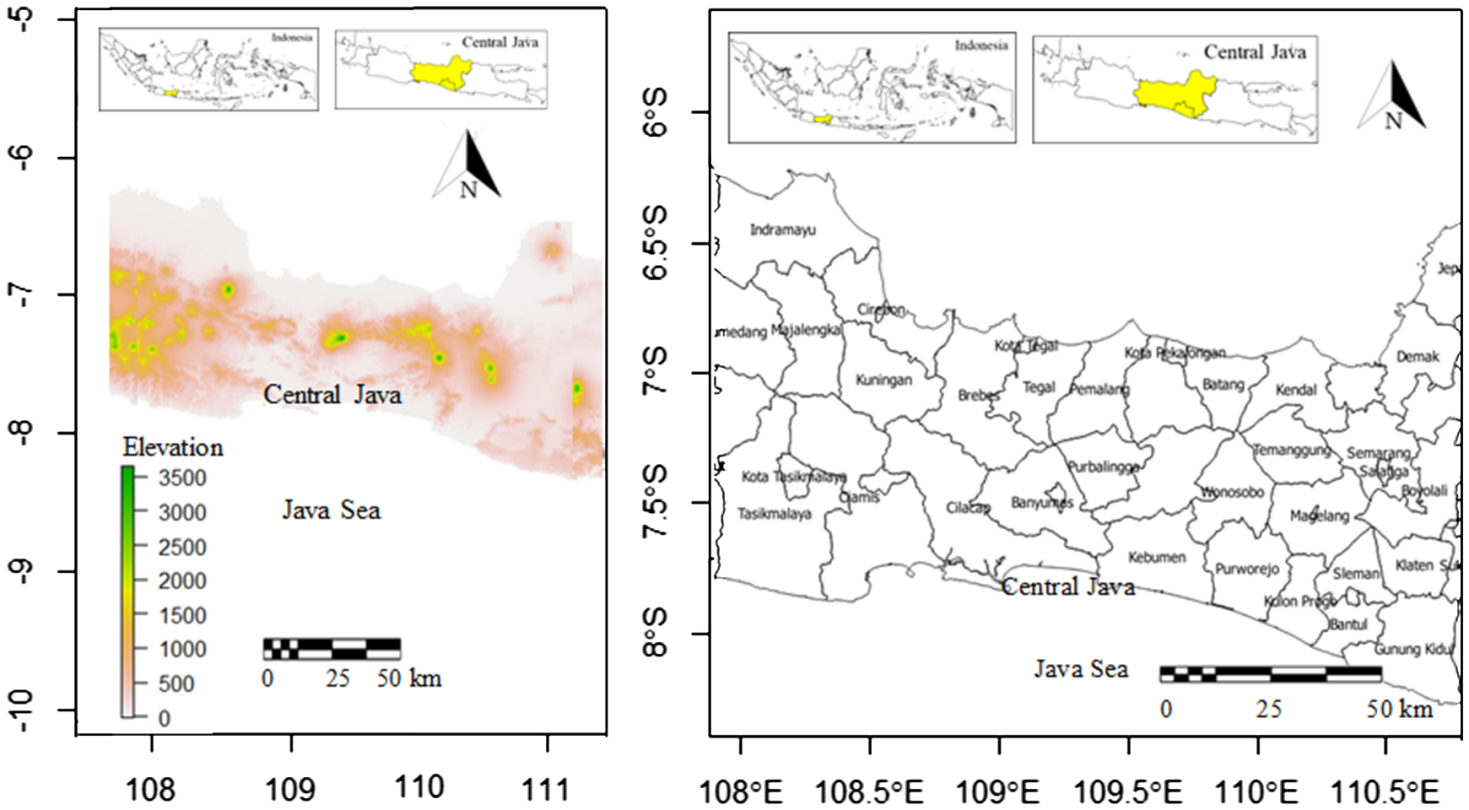
Locations and elevations (m) of study areas in Central Java Province, Java Island, Indonesia (left) and districts in study areas (right).

The study area was Central Java Province located in 108.00^0^ - 111.00^0^ east longitude and 6.00^0^ – 8.00^0^ south latitude (**Figure 1**). Central Java sizing 32,800.69 km^2^ is largely hilly especially in the middle. The slope level of land in Central Java is as follows: 38% has a slope of 0-2%, 31% has a slope of 2-15%, 19% has a slope of 15-40%, and the remaining 12% has a slope of more than 40%. Central Java is primarily an agricultural region. Wet rice is the main food crop in here. Other crops farmed on small landholdings in lowland areas include corn (maize), cassava, peanuts (groundnuts), soybeans, and sweet potatoes. Terraced hillslopes and irrigated paddy fields are common landscape characteristics. For local consumption, kapok, sesame, vegetables, bananas, mangoes, durian fruits, citrus fruits, and vegetable oils are grown. Exports include tea, coffee, tobacco, rubber, sugarcane, kapok, and coconuts. The average temperature of Central Java is between 18 and 28°C, with a relative humidity of 73% to 94%. While humidity is high in most of the province’s low-lying areas, it lowers dramatically in the highest highlands. Salatiga in the middle had the highest average annual rainfall of 3990 mm, with 195 rainy days. This study covers numerous districts (**Figure 1**), including Indramayu, Sumedang, Majalengka, Cirebon Demak, Kendal, Batang, Semarang, and Pekalongan Districts, representing northern coastal areas, and Tasikmalaya, Ciamis, Cilacap, Kebumen, Purworejo, Kulon Progo, and Bantul, representing southern coastal areas. The interior of the studied areas, dominated by hillslopes, is represented by Boyolali, Salatiga, Magelang, Temanggung, Wonosobo, Purbalingga, Brebes, and Kuningan Districts.

The topography of Central Java is available in **Figure 1**. It is clear that most of the middle parts of Central Java were dominated by mountain ranges with elevation ranges of 1500 – 3500 m. The north was dominated by the shore, and more hilly areas dominated the south of the middle parts of Central Java. The western parts have more mountainous areas in comparison to the eastern parts. The south was bordered by Java Sea.

### 2.2 *Artocarpus altilis* occurrence surveys

**Figure 2:**
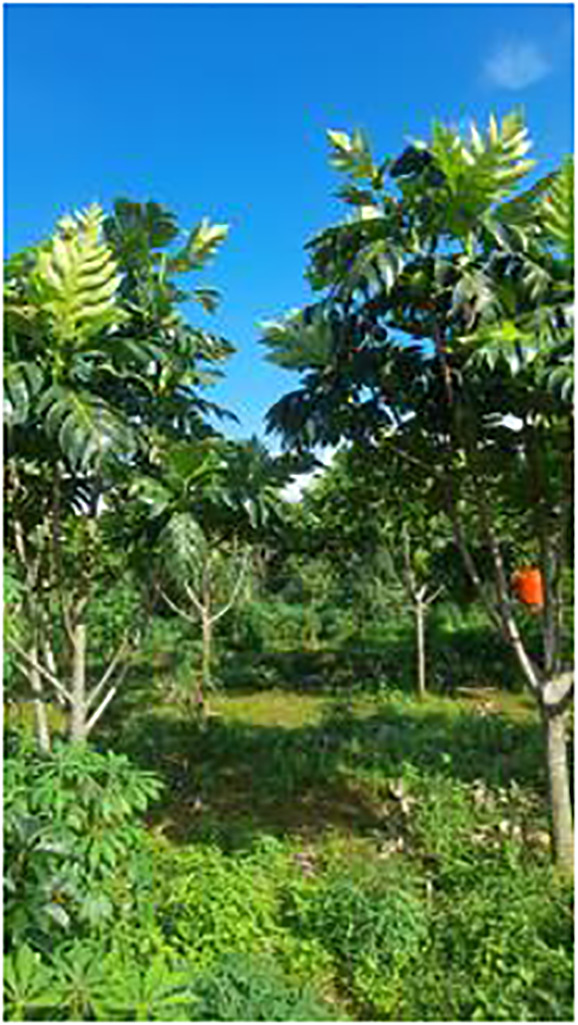
*A. altilis* crop trees in Central Java Province, Java Island, Indonesia (Photo: Suyud Warno Utomo).

Explorations or crop field surveys (Scrivanti and Anton, 2020; Gufi et al., 2023) were conducted in all areas of Central Java, Indonesia, to record the existence *of A. altilis* (**Figure 2**) in real time. The field location was chosen using information from the Herbarium Bogoriense and a database provided and gathered from literature reviews and the Agency for Agriculture and Forestry of Ministry for Agriculture and Forestry, Indonesia. The geographical coordinates of *A. altilis* occurrences in the field were recorded using the Garmin Etrex 30 type Global Position System (GPS). The data were converted into Microsoft Excel and saved in CSV format for use in SDM habitat suitability modeling.

### 2.3 Bioclimatic variables

This study included various bioclimatic variables (**Table 1**) following Dong et al. (2023) and Arshad et al. (2022). For the recent time, bioclimatic variables (Bio 1 – Bio 19) were retrieved from the global climate database WordClim (www.worldclim.org, the new version 2.0) (Hijmans et al., 2005). This database has been employed extensively in habitat suitability modeling (Khanum et al., 2013) and widely used in the Asian region (Rana et al., 2017). Bioclimatic variables were visualized as grids. These grids commonly referred to as 1-km spatial resolution and have a 30 arc-seconds spatial resolution equals to about 0.86 km*^2^* at the equator and less elsewhere (Fick and Hijmans, 2017).

**Table 1:** Bioclimatic variables used in this study (Ulak and Paudel, 2021).

Variables	Sources	Format	Unit
Annual mean temperature (Bio 1)	www.worldclim.org	Image data in Raster	^0^C
Mean diurnal range (Bio 2) (mean of monthly (max temp - min temp))	www.worldclim.org	Image data in Raster	^0^C
Isothermality (Bio 3)*	www.worldclim.org	Image data in Raster	%
Temperature seasonality (Bio 4)*	www.worldclim.org	Image data in Raster	^0^C
Max temperature of warmest month (Bio 5)	www.worldclim.org	Image data in Raster	^0^C
Min temperature of coldest month (Bio 6)	www.worldclim.org	Image data in Raster	^0^C
Temperature annual range (Bio 7)	www.worldclim.org	Image data in Raster	^0^C
Mean temperature of wettest quarter (Bio 8)	www.worldclim.org	Image data in Raster	^0^C
Mean temperature of driest quarter (Bio 9)	www.worldclim.org	Image data in Raster	^0^C
Mean temperature of warmest quarter (Bio 10)	www.worldclim.org	Image data in Raster	^0^C
Mean temperature of coldest quarter (Bio 11)	www.worldclim.org	Image data in Raster	^0^C
Annual precipitation (Bio 12)	www.worldclim.org	Image data in Raster	mm
Precipitation of wettest month (Bio 13)	www.worldclim.org	Image data in Raster	mm
Precipitation of driest month (Bio 14)	www.worldclim.org	Image data in Raster	mm
Precipitation seasonality (Bio 15) *	www.worldclim.org	Image data in Raster	dimensionless
Precipitation of wettest quarter (Bio 16)	www.worldclim.org	Image data in Raster	mm
Precipitation of driest quarter (Bio 17)	www.worldclim.org	Image data in Raster	mm
Precipitation of driest quarter (Bio 18) *	www.worldclim.org	Image data in Raster	mm
Precipitation of coldest quarter (Bio 19) *	www.worldclim.org	Image data in Raster	mm

*: selected variables based on multicollinearity test.

Those bioclimatic variables were chosen based on selection and utilization of bioclimatic variables having a significant influence in order to obtain an accurate and informative habitat suitability model. Jackknife analysis (gam_jacknife package) was used to evaluate the contribution of each bioclimatic variable to the resulting model. Some bioclimatic variables were not used due to the lack of contribution to the model making (percent contribution = 0). Those bioclimatic variables were variables with a small average contribution (<6%) or permutation importance (<6%) (Wei et al., 2018). The contribution percentage and permutation are two important factors for understanding and measuring the bioclimatic variable’s contribution as well as importance to the machine learning, geoclimate, and statistical based model.

### 2.4 Multicollinearity test

To establish a model that has better performance with fewer variables and to avoid collinearity between the variable, a multicollinearity test using mctest package (Imdadullah et al., 2016) was performed using Pearson’s correlation tests (Préau et al., 2018) on 19 bioclimatic variables (Bio 1 – Bio 19). The variables that have highly cross-correlated variables (r^2^ > 0.8) were excluded and variables having r^2^ < 0.8 were kept for further analysis for geographical distribution modeling. If multicollinearity occurs, then a variable is strongly correlated with other variables in the model, and its predictive power is unreliable and unstable (As’ary et al., 2022). Based on the multicollinearity test, the selected environmental variables to be used were Bio 3, 4, 15, 18, and 19 (**Table 1**).

### 2.5 SDM and climate change RCP analyses

This study employed machine learning, geoclimate, and statistical based analysis using species modeling packages (**Table 2**) (Dolci and Peruzzi, 2022) within R platform version 3.6.3 (Mao et al., 2021) to generate predicted suitability cultivation areas of breadfruit across Central Java, Java Island, Indonesia. Several R packages required to develop the suitability maps include library (“sp”), library (“dismo”) (Khan et al., 2022), library (“rgdal”) (Bivand, 2022). and library (“raster”) (Lemenkova, 2020). SDM analysis is using dismo package. The inputs for model included Bio 3, 4, 15, 18, and 19 as selected variables.

**Table 2:** Evaluated climate change variables in this study.

Variables	Sources
Current climate	www.worldclim.org Bio 1 – Bio 19 with the resolution of 30 arc-seconds, ~1 km at the Equator (Navarro-Racines et al., 2020).
Future climate	CMIP5 RCP 2.6, RCP 8.5 for 2050 and 2070 with the resolution of 2.5 arc-minutes, ∼5 km) (Hussain and Dhiman, 2022).

Within the model, the contribution and impact of each bioclimatic variable on the breadfruit suitable cultivation areas model were determined using a jackknife test K (Promnikorn et al., 2019), and the receiving operating curve (AUC) area was used to evaluate the performance model. According to Zhu et al. (2017), AUC values range from 0 (least appropriateness) to 1, with a value less than 0.5 indicating that the resultant model is not better than random and uninformative data, and a value greater than 1.0 indicating that the resulting model is highly good and informative.

Following that, the analysis findings from machine learning models predicting breadfruit suitable cultivation area ranges were imported into GIS for presentation and additional study (Hijmans et al., 2012). The QGIS platform version 2.16 was used in this study. According to Wei et al. (2018), habitat suitability levels on the employed machine learning model map can be classified into five suitability level included 0: unsuitable, 0-1: low suitability, 1-2: medium suitability, 2-3: high suitability, 3-4: very high suitability.

This study’s climate change analyses were based on Representative concentration pathways (RCPs). RCPs are four greenhouse gas concentration and not emission trajectories, according to the Intergovernmental Panel on Climate Change (IPCC) in its AR5 in 2014 (IPOC, 2008). This replaces the forecasts in the Special Report on Emissions Scenarios (SRES) issued in 2000 (Vuuren et al., 2009). These Coupled Model Inter-comparison Project-phase 5 (CMIP5) pathways are used in climate modeling and research to represent four possible future climates, all of which are deemed possible depending on how much greenhouse gas is emitted in the near future. The four RCPs are called after a hypothetical range of Radiative Forcing values in the year 2100 relative to pre-industrial levels ranging from + 2.6, + 4.5, + 6.0, to + 8.5 W/m^2^ (Weyant et al., 2009). RCP2.6 is optimistic scenario estimating about 1°C increase before the end of XXI century, RCP4.5 is moderate scenario estimating 1.8°C increase, and RCP8.5 is pessimistic, scenario estimating about 3.7°C increase (Collins et al., 2013; Alipour and Walas, 2023). Here, we selected the RCP2.6 as the minimum emission representative and RCP8.5 as the maximum emission representative models to simulate habitat suitability distributions of *A. altilis* in the 2050s and 2070s (Beaumont et al., 2008). SDM output for crop cultivation area suitability distribution of *A. altilis* were reclassified in QGIS with spatial analyst to reclassify and calculate the sizes of suitable cultivation areas in km^2^ based on five suitability levels.

## 3 Results

### 3.1 Model evaluation and validation

Model evaluation and validation guarantee the reliability of modeling results, as expressed by the area under the receiver-operating characteristic (ROC) curve (AUC) obtained by the accuracy test of the ROC curve analysis method. The AUC values were between 0 and 1 and divided into several value classes. When the AUC value was lower than 0.5, the model executed was worse than contingency. When the AUC value ranges from 0.5 to 0.6, the model performance is considered poor; 0.6–0.7 is considered fair; 0.7–0.8 is considered good; 0.8–0.9 is considered very good; and 0.9–1 is considered excellent. The closer the AUC test value is to 1, the better the discrimination and the more precise and descriptive the model.

In this study, as can be seen in **Table 3**, all climate scenarios have the AUC greater than 0.8, among which the RCP8.5 model has the highest values. The lowest AUC value was recorded for current scenario model and RCP2.6 for 2050. The AUC results suggest that the model’s prediction accuracy could be rated as excellent. This result can therefore be used to identify the best models to represent the cultivation area suitability of breadfruit in Central Java.

**Table 3:** AUC values of current and future climate scenario models for breadfruit crops *Artocarpus altilis* in Central Java, Indonesia.

Scenarios	UC values
Current	0.852
RCP 2.6 for 2050	0.895
RCP 2.6 for 2070	0.915
RCP 8.5 for 2050	0.930
RCP 8.5 for 2070	0.930

### 3.2 Contribution of bioclimatic variables to the suitable cultivation areas of *Artocarpus altilis* in Central Java, Indonesia

The multicollinearity tests have resulted in selecting five determinant bioclimatic variables including Bio 3, 4, 15, 18, and 19. Those selected bioclimatic variables have varied contribution to the suitable cultivation areas of *Artocarpus altilis* in Central Java, Indonesia as can be seen in **Table 4**. From the results, precipitation seasonality followed by the precipitation of driest quarter was the bioclimatic variables that have the most contribution to the suitable cultivation areas of breadfruit with the values of 42.24% and 25.28%. While precipitation of coldest quarter followed by isothermality with the values of 2.29% and 9.19% have the lowest contribution to the distributions of suitable cultivation areas (**Table 4**).

**Table 4:** Relative contributions of selected climatic variable to the SDM model for breadfruit crops *Artocarpus altilis* in Central Java, Indonesia.

Variables	Descriptions	Contribution (%)
Bio3	Isothermality (Bio 3)	9.19
Bio4	Temperature seasonality (Bio 4)	20.97
Bio15	Precipitation seasonality (Bio 15)	42.24
Bio18	Precipitation of driest quarter (Bio 18)	25.28
Bio19	Precipitation of coldest quarter (Bio 19)	2.29

### 3.3 Response curves of bioclimatic variables

**Figure 3:**
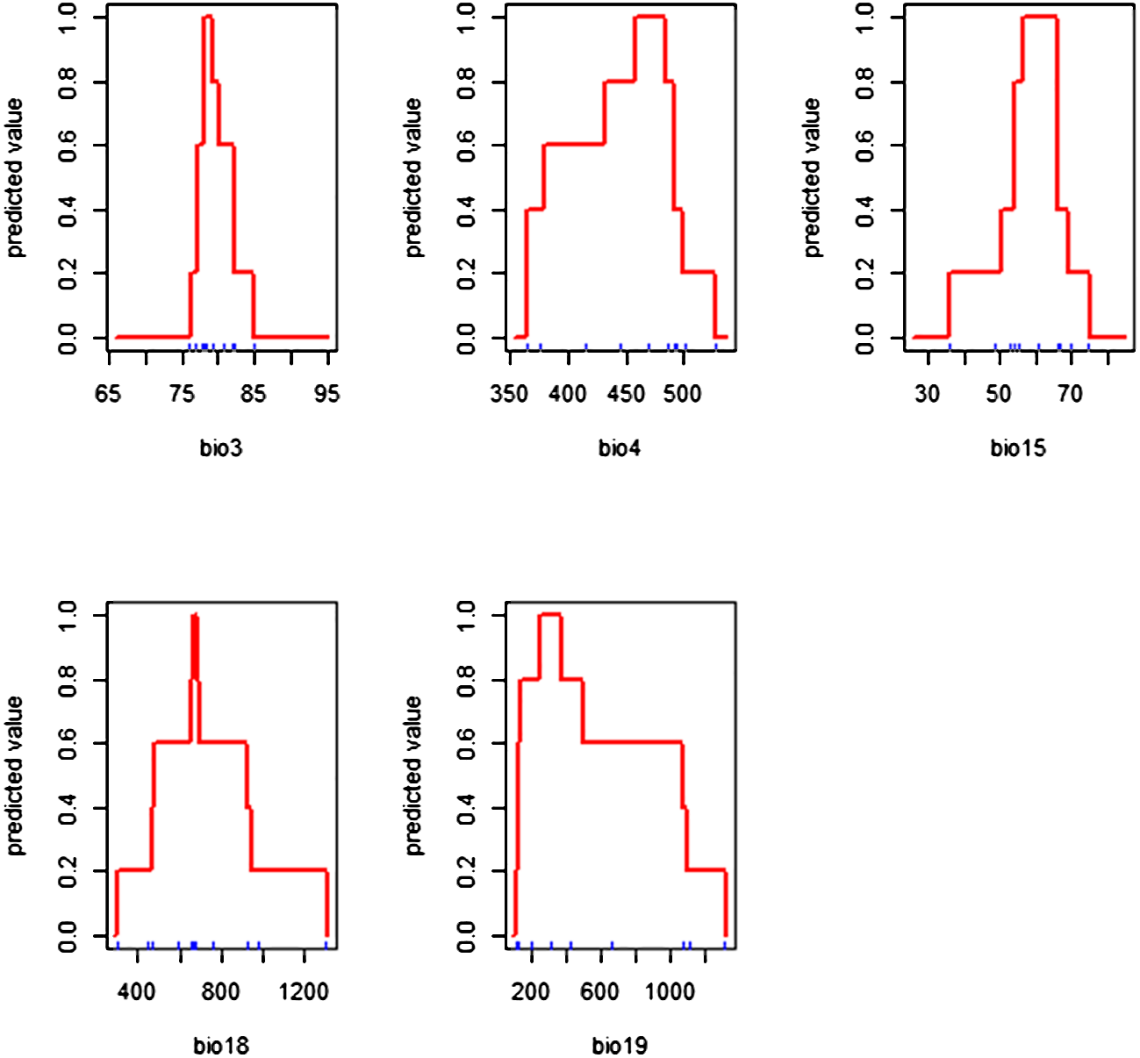
Response curves of *Artocarpus altilis* to the selected bioclimatic variables.

The response curves show the relationships between probability of occurrence and habitat suitability level of breadfruit with each bioclimatic variable (**Figure 3**). The response curves of the suitable cultivation areas of breadfruit were drawn for the selected five bioclimatic factors. The existence probability of each bioclimatic factor to breadfruit showed that with the increase in the bioclimatic factor value, the existence probability showed a trend of first increasing rapidly and then decreasing slowly. In this study, the range of bioclimatic factors when the probability of suitability is greater than 0.6 was used to represent the climate characteristics of the breadfruit the suitable cultivation areas. The climate characteristics of the distribution area of breadfruit are as follows: the isothermality (Bio 3) is 77–82%, the temperature seasonality (Bio 4) is 37.0–47.0°C, the precipitation seasonality (Bio 15) is 55–65, the precipitation of driest quarter (Bio18) is 50-90 mm, and the precipitation of coldest quarter (Bio19) is 10-45 mm.

### 3.4 Potential cultivation areas under current climate

**Figure 4:**
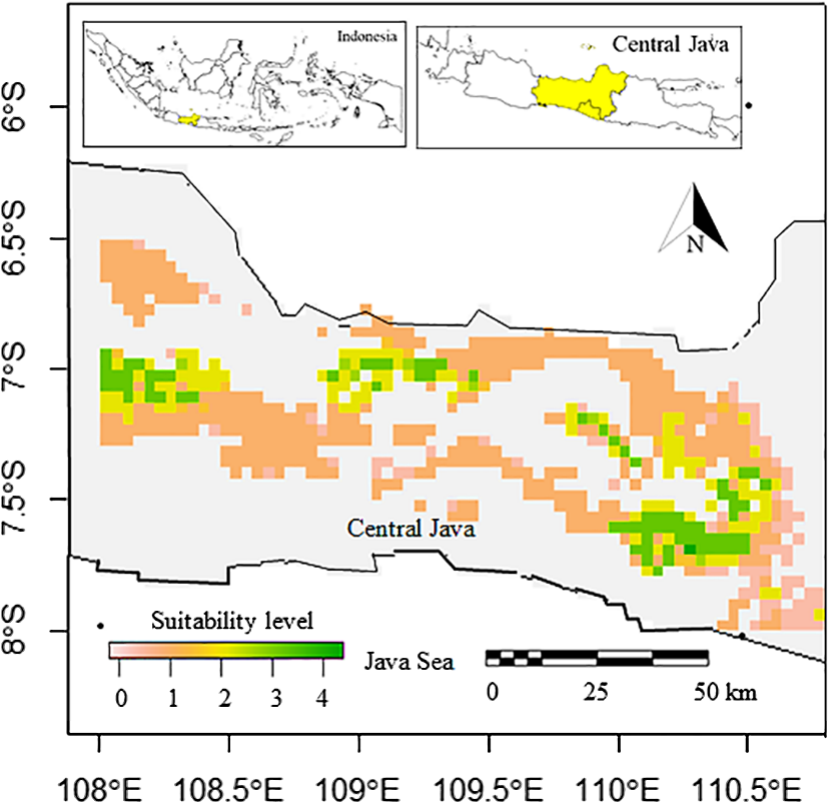
Current suitable cultivation areas of breadfruit crops *Artocarpus altilis* in several districts in Central Java, Indonesia (0-1: low suitability, 1-2: medium suitability, 2-3: high suitability, 3-4: very high suitability).

The potentially suitable cultivation areas of breadfruit are currently mainly distributed on the north, east, west, and south of Central Java, except in the central parts. In the west, districts classified as having very high suitability are observed in Sumedang and Majalengka, Brebes and Tegal in the north, Wonosobo, and Temanggung in the east. Large, very suitable areas are observed in the southeast, covering Magelang, Purworejo, Sleman, Boyolali, and Kulon Progo Districts. Districts located in the central parts rarely had suitable areas. The central parts of Central Java were characterized by highlands and mountainous ranges. Based on the model, this highland was not suitable for cultivating breadfruit (**Figure 4**). Based on the calculation, as can be seen in **Table 5**, suitable cultivation areas for breadfruit account for 9737.778 km^2^, or 28.35% of Central Java’s total area. Within size, areas categorized as high suitability were dominant and accounted for 6282.672 km^2^.

**Table 5:** Current and under 2070 RCP 8.5 scenario suitable cultivation areas in km^2^ of breadfruit crops *Artocarpus altilis* in Central Java, Indonesia, (1: low suitability, 2: medium suitability, 3: high suitability, 4: very high suitability levels).

Suitability level	Current	2050		2070	
		RCP 2.6	RCP 8.5	RCP 2.6	RCP 8.5
4	1514.666	400	388.571	450	461.571
3	1548.466	2823	2286.133	3359	3326.133
2	6282.672	1323	1600.329	787	675
1	391.974	0	0	0	0
Total	9737.778	4546	4275.033	4596	4462.704
Percentage of total areas	28.35%	13.23%	12.45%	13.38%	12.99%

### 3.5 Potential cultivation areas under climate change in 2050

**Figure 5:**
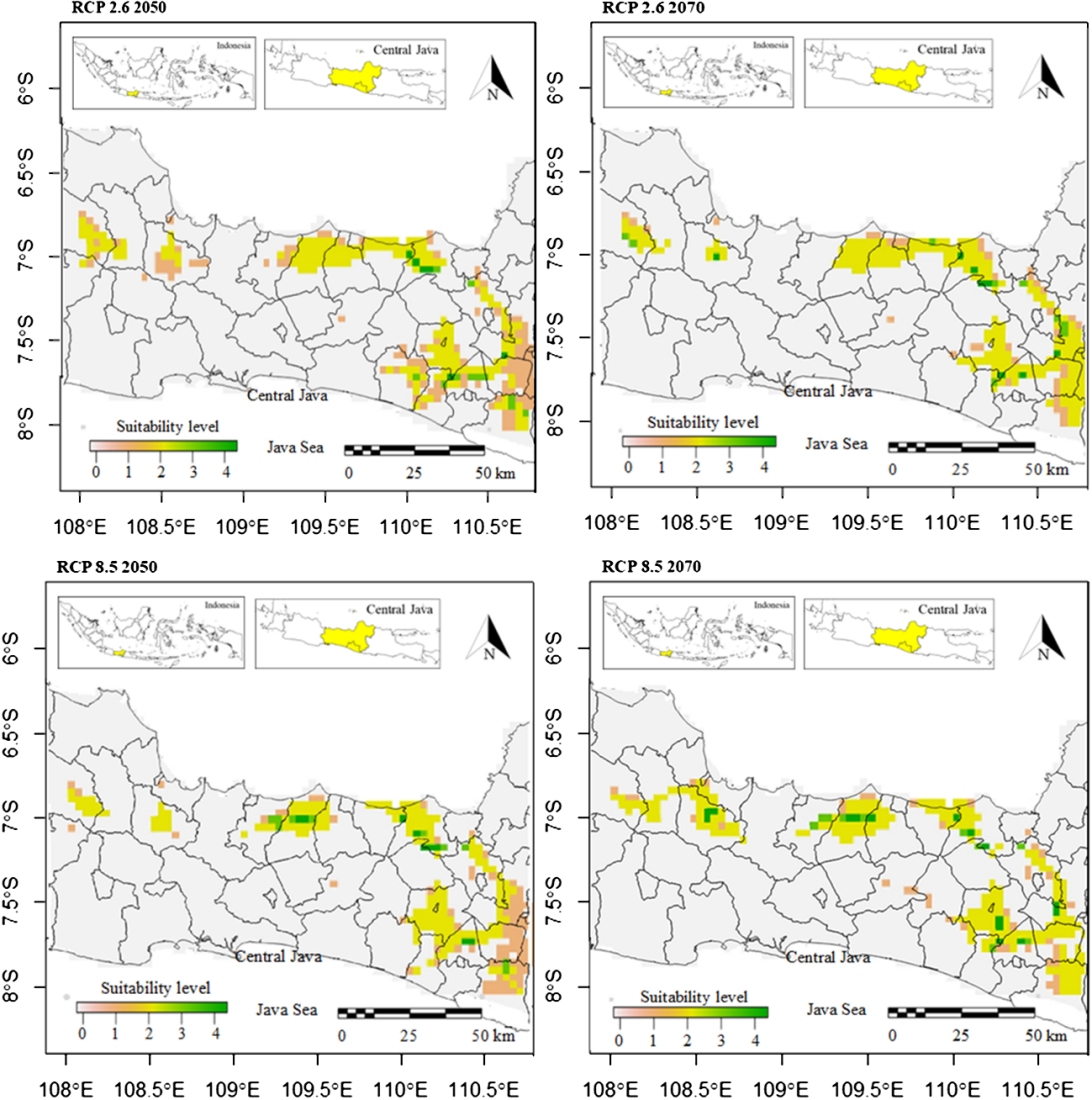
Future suitable cultivation areas of breadfruit crops *Artocarpus altilis* in several districts in Central Java, Indonesia under current and future 2050-2070 RCP2.6 and 2050-2070 RCP8.5 scenarios (0-1: low suitability, 1-2: medium suitability, 2-3: high suitability, 3-4: very high suitability).

The potentially suitable cultivation areas for breadfruit in 2050 were estimated to be reduced. This reduction was observed for both optimistic scenario and pessimistic scenarios. Under optimistic scenarios (**Figure 5**; **Table 5**) or known as RCP 2.6, the suitable cultivation areas currently were only available for 13.23%. The suitable areas were only limited to north, east, and southeast parts of Central Java. All districts previously suitable for breadfruit in the west were not suitable and shifted eastward to the Kendal District on the north coast and Kulon Progo District in the southeast. While, under pesimistic scenarios or known as RCP8.5, the suitable cultivation areas currently were only available for 12.45% or lower than optimistic RCP2.6 scenario. The suitable areas were only limited to north and east parts of Central Java. For both scenarios and contrast to current scenarios, areas categorized as medium suitability were dominating and larger than other suitability levels. Implementing mitigation climate change scenario and limit the temperature increase to only 1°C will provide 270.967 km^2^ more of suitable cultivation areas for breadfruit.

### 3.6 Potential cultivation areas under climate change in 2070

**Figure 6:**
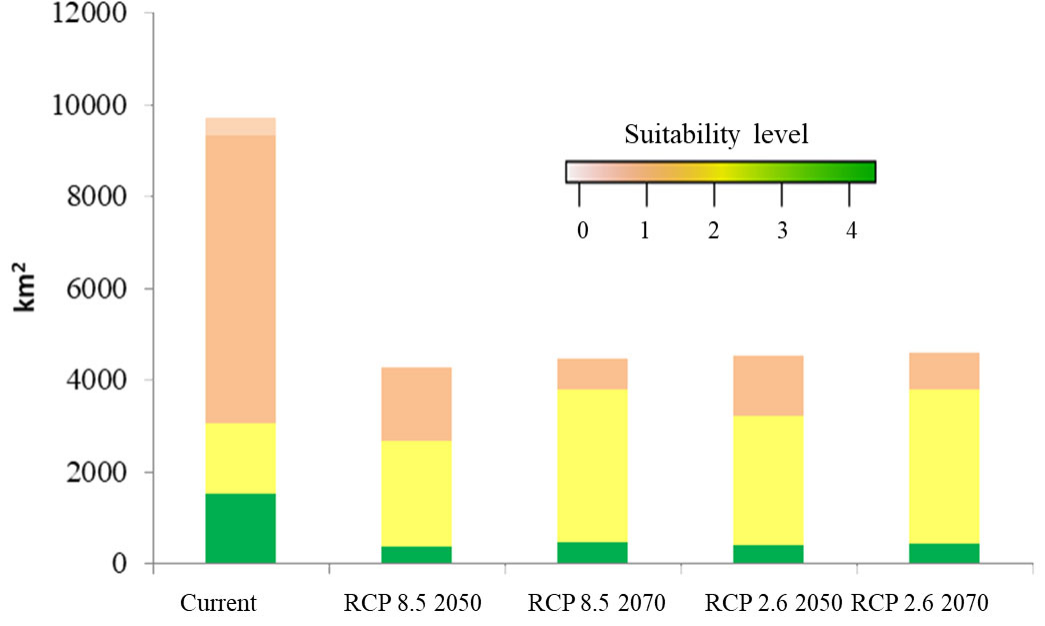
Compositions of suitable cultivation areas of breadfruit crops *Artocarpus altilis* in Central Java, Indonesia under current and future 2050-2070 RCP2.6 and 2050-2070 RCP8.5 scenarios (0-1: low suitability, 1-2: medium suitability, 2-3: high suitability, 3-4: very high suitability).

The potentially suitable cultivation areas for breadfruit in 2070 were estimated to increase. This increase was observed for both optimistic and pessimistic scenarios. Under optimistic scenarios (**Figure 5**; **Table 5**) or known as RCP2.6, the suitable cultivation areas are currently only available for 13.38% or will increase up to 50 km^2^ by 2050. While, under pessimistic scenarios, known as RCP8.5, the suitable cultivation areas are currently only available for 12.99% or lower than the optimistic RCP2.6 scenario. The suitable areas were only limited to the north and east parts of Central Java. Kulon Progo District in the southeast, which was previously considered suitable for breadfruit in 2050, is now not considered suitable in 2070. Under RCP8.5, the suitable cultivation areas increased by 187.671 km^2^. Implementing a mitigation climate change scenario and limiting the temperature increase to only 1°C will provide 133.296 km^2^ more of suitable cultivation areas for breadfruit in 2070. In the year 2070, for both scenarios, the medium suitable areas were reduced and replaced with high suitable areas (**Figure 6**). This condition was observed in the eastern parts of Central Java. Highlands in Boyolali that were suitable for breadfruit under the current scenario now, in 2070, become suitable again. Under the optimistic model, high suitability areas increased up to 536 km^2^ and 1040 km^2^ for the pessimistic model, replacing the medium suitability areas in the east parts.

## 4 Discussions

The *A. altilis* presences recorded during the surveys were parts of the agroforestry systems. This finding is in agreement with previous study that clarifies agroforestry are crucial for the breadfruit cultivation practices (Siarudin et al., 2021). In this study, the method to assess the potential cultivation areas for expanding breadfruit is using SDM. The use of this method contributed to better conserve breadfruit habitat using the following approach, first it provided key bioclimatic variables highly correlated with breadfruit distribution. Second, SDM model was developed to quantify the relationship between breadfruit presence and the selected bioclimatic variables including its inverse variables, as reported in this study was climate change (Yi et al., 2016). The uses of SDM in this study to assess the impacts of climate changes on the potential distribution of vegetation were in agreement with the previous study (Ali et al., 2023; Daï et al., 2023).

This study confirmed that Bio 3, Bio 4, Bio 15, and Bio 18 are some fundamental bioclimatic variables shaping the tree species distribution, as reported before (Ali et al., 2023). Those bioclimatic variables were related to the temperature and precipitation aspects. The roles of these climatic variables are very prominent in the distribution of breadfruit in Central Java. Precipitation in the warmest quarter was detected as the leading variable in the distribution of tree species (Rathore et al., 2019). Similarly, isothermality represents the proportion of the mean diurnal range to the annual temperature range. Hence, day and night and summer and winter temperature differences strongly influence the considered tree species. This means that the predicted eastward niche shift for breadfruit in the Central Java region would be chiefly influenced by temperature and precipitation variations. Similar findings were also reported by Su et al. (2005) and Li et al. (2019), who communicated the role of temperature and precipitation factors in tree species distribution. Breadfruit-suitable cultivation areas are expected to expand due to precipitation. Increased precipitation during the driest quarter and overall increases in annual precipitation cause the prospective expansion of suitable breadfruit areas at the periphery of the baseline acceptable range (Zhao et al., 2020; Yang et al., 2022).

Temporal and climate change dimensions can have potential impacts on vegetation spatial distributions. The impacts can increase the size of distributions or reduce them. In this study, the temporal variation causes an increase in suitable cultivation areas by the year 2070 for both climate change scenarios. By comparing the emission strategies, lowering the emissions will lead to more suitable cultivation areas. This finding confirming the implementation of RCP2.6 will increase suitable areas and RCP8.5 will cause decline was in accordance with the precious study on vegetation (Yu et al., 2023) and particular breadfruit (Yang et al., 2022). According to recent studies, in Southeast Asia, 80.7% of the region, or 3.6 million km^2^, is considered good for breadfruit, while 77.4%, or 2.8 million km^2^, is considered fair. A recent study also confirmed the adaptability of breadfruit against climate change scenarios. In the Southeast Asia region, the breadfruit-suitable area primarily persists and even increases by 0.7%, or equal to 23000 km^2^, under the RCP2.6 scenario and by 0.8%, or 30000 km^2^, under the RCP8.5 scenario. A study from the tropical Hawaii Islands (Mausio et al., 2020) confirmed successive increases in breadfruit suitability in the RCP4.5 and RCP8.5 scenarios, with an increase of 27% for RCP4.5 and 89% and for RCP8.5, respectively. Then, it concludes that breadfruit is a potential crop at least until 2070 that can adapt to climate change situations in which the optimum results can be gained by investing more in climate change mitigation strategies.

The shifting of breadfruit to the east under climate change, where eastern parts of central Java have higher temperatures, is in agreement with the current predictions. Breadfruit is considered a crop that can deal with the warming environment due to climate change. However, the potential areas are fragmented. Despite the fact that the east and southeast of central Java are predicted to provide suitable habitats for breadfruit, this area is dominated by mountain ranges. These mountain ranges can provide physical barriers to breadfruit seed dispersal (Qiong et al., 2017). Despite that, the mountain environment has specific local climate characteristics (Porceddu et al., 2020) that limit the presence of breadfruit in some mountainous areas in central Java. The shifting of breadfruit to the east, followed by a reduction some potential habitats, is in agreement with previous studies. In low-altitude areas that are located in the subtropical and tropical regions, climate change will significantly contribute to the reduction of suitable areas of staple crops compared to the crops raised in the higher latitudes (Levis et al., 2018).

The shifting of breadfruit expansions as modeled in this study will lead to ecological and socioeconomic impacts. The model shows that some areas that were previously suitable for breadfruit will disappear, especially in the western region. The reduction of suitable areas will then impose food security in certain areas. In contrast, the expansion of breadfruit eastward will provide opportunities for using breadfruit as an alternative crop in the eastern regions of Southeast Asia. This will mitigate the food security issues in those areas.

### 4.1 Limitations of this study

In this study, we selected Central Java as the study area, considering that the *A. altilis* grown in this area has been recognized as an agricultural geographical indication product by the Ministry of Agriculture of Indonesia’s Government, and the high quality of breadfruit grown in this area has been recognized by the market and community. Despite the fact that this study has identified a vast potential area suitable for breadfruit, the Central Java areas were threatened by land conversions and deforestations (Prasetyo et al., 2013), which resulted in potential agricultural lands being converted into settlements. This study does not include the land conversion variables. This may result in a vast potential area for breadfruit, which may be reduced significantly due to those land conversions.

## 5 Conclusion

In this study, we modeled the current spatial distribution of *A. altilis* potential cultivation areas, tested the key bioclimatic indicators combined with climate change that could potentially affect distribution, and simulated its suitable cultivation areas. Our results show that the potential cultivation areas of *A. altilis* follow mostly the precipitation seasonality (Bio 15), precipitation of the driest quarter (Bio 18), and temperature seasonality (Bio 4) gradients. As hypothesized by previous studies, the spatial distribution of *A. altilis* has the potential to expand by the year 2070 despite its area reductions in the current climate. As can be seen under both optimistic and pessimistic scenarios, the suitable cultivation areas categorized as having high suitability will expand eastward. These expansions will add 536 km^2^ of high-suitability areas under RCP2.6 and 1040 km^2^ for RCP8.5 in 2070. Implementing a mitigation climate change scenario and limiting the temperature increase to only 1°C will provide 270.967 km^2^ more of suitable cultivation areas for breadfruit in 2050 and 133.296 km^2^ in 2070.

The model indicating the potential habitats for breadfruit that are resilient to climate change is mostly in the east of central Java. Then, to anticipate this, the local agriculture authorities are urged to have some preparation. Considering that Java Island is threatened by rapid deforestation and land use changes, regions in the eastern parts considered to have high suitability should be protected immediately from logging, mainly in Kendal District, which is predicted to have the most suitable habitats under climate change. Due to the fact that intensive breadfruit plantations may also lead to logging, alternative solutions should be considered. One alternative that can be taken is agroforestry. In this scenario, breadfruit is grown together with the native woody species.

## Data availability statement

The original contributions presented in the study are included in the article/supplementary material. Further inquiries can be directed to the corresponding author.

## Author contributions

SU: Conceptualization, Writing – review & editing. FL: Conceptualization, Writing – review & editing. AA: Software, Writing – original draft. F: Conceptualization, Writing – review & editing. MRF: Conceptualization, Writing – review & editing. HQ: Project administration, Writing – review & editing.
